# Reviewing the preclinical curriculum in a Problem Based Learning driven medical program: challenges and strategies

**DOI:** 10.15694/mep.2019.000005.1

**Published:** 2019-01-09

**Authors:** Manori Amarasekera, Paul S Noakes, Brian D Power

**Affiliations:** 1 School of Medicine Fremantle; 2 School of Medicine Fremantle

**Keywords:** Curriculum review, preclinical, education, curriculum, problem based learning, medical education, constructive alignment, teaching and learning

## Abstract

This article was migrated. The article was marked as recommended.

In order for medical curricula to remain progressive and contemporary, continuous review is critical to ensure that the learners are directed to achieve the intended goals and become workforce ready. We developed a framework for continuous curriculum review at the School of Medicine Fremantle (The University of Notre Dame Australia), taking the key aspects of a curriculum review process into consideration. In planning and implementing the review process we identified several challenges, including management of metadata, work load on staff members, and evaluation. These challenges were addressed successfully by applying necessary strategies using limited resources. The framework we have developed provides a guide to key stakeholders who are involved in medical curriculum review and development.

## Introduction

Given the changing landscape in medical education (whether from the establishment of new medical schools, a shift in health care needs and demographics requiring a shift in medical force training, or the ever-changing landscape of educational approaches in adult learning) ( Duckett 2005; Hays 2016), ongoing review of the medical curriculum remains core business of any medical school. Curriculum review may occur in response to a specific issue (i.e., for the purposes of accreditation) or is a continuous process embedded in quality assurance systems ( Goulston and Oates 2008; Mwakigonja 2016).

Reviewing the medical curriculum that occurs in response to a particular issue is associated with well-defined goals and frameworks and often based on protocols developed by accreditation agencies, such as the Australian Medical Council ( Goulston and Oates 2008). This is in contrast to continuous curriculum review that is undertaken mainly to maintain and refresh the curriculum. As continuous curriculum review is not necessarily based on national protocols and accreditation standards, and the medical curriculum is significantly different across universities both globally and nationally, it becomes challenging to develop a highly effective framework for the review process.

This paper discusses some of the many challenges that were encountered throughout the process of planning and implementing formal review and development processes of the preclinical curriculum at the School of Medicine Fremantle (The University of Notre Dame Australia, SOMF) and some strategies adapted to successfully achieve the targeted outcomes of this continuous process. We believe that in sharing our local experience this might assist the wider community of medical educators and university stakeholders, firstly, in designing their medical curriculum review and secondly, in encouraging others to explore new strategies for overcoming many of the challenges faced in reviewing the curriculum.

## Preclinical medical curriculum of the School of Medicine Fremantle (The University of Notre Dame Australia)

The preclinical curriculum is delivered in first and second years of the four year Doctor of Medicine (MD) program at SOMF. While the preclinical curriculum is based heavily on basic and clinical sciences, it is an integrated curriculum from the outset, with four core domains, {basic and clinical sciences (BCS), clinical and communication skills (CCP), personal and professional development (PPD) and population and preventative health (PPH)} and aboriginal health (AH) and Research (R) where students are introduced to the knowledge, skills, attitudes and values deemed essential as a junior doctor working in the Australian health system. As students progress through the curriculum, they revisit previous learning to re explore and extend it, with each step taking students to a more complex stage of the original concept (spiral learning) ( Masters and Gibbs 2007; Laskaratos et al. 2014).

Problem-based Learning (PBL) continues to be the pedagogical model of teaching and learning in the preclinical years and basic sciences and clinical scenarios are ‘problem-solved’ in facilitated small group tutorials. This approach sits comfortably in the weekly timetable alongside the delivery of other educational strategies (lectures, laboratory classes, clinical skills tutorials, etc.) and resourcing is shared between the main campus in Fremantle and a partner institution (Murdoch University), a factor which had to be taken into consideration when reviewing the preclinical curriculum at SOMF.

**Figure 1.**
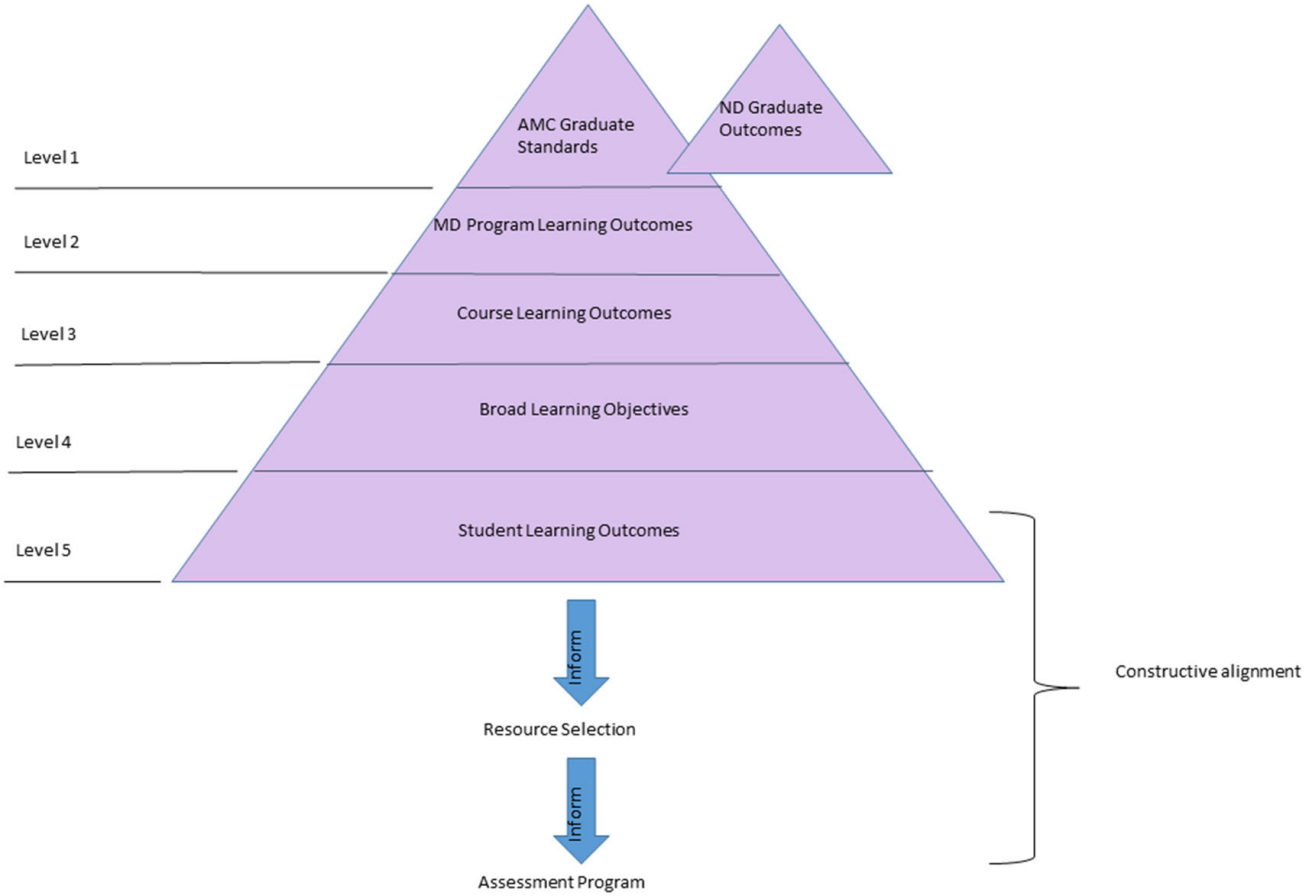
Hierarchical organization of outcome statements in the SOMF curriculum framework.

Adapted from “Prudentia: A medical school’s solution to curriculum mapping and curriculum management” by Steketee C. 2015. *Journal of University Teaching and Learning Practices, 12(4).*

The PBL tutorials are organized into ‘blocks’ based on the discipline or the body systems and are ordered in a manner that sequentially introduces basic to more complex concepts over time. Some blocks appear in both the first and second year curriculum facilitating vertical integration, a key principle underlying the existing preclinical curriculum. Within the school’s curriculum framework the hierarchical relation of different levels of learning outcomes ( Figure 1) has a significant effect on the constructive alignment ( Steketee 2015) and has informed the design of our curriculum review process.

## Developing a framework for the preclinical curriculum review process

The objectives of the curriculum review at the SOMF is to augment the existing areas of strength in the preclinical curriculum and through innovation, address the gaps in the learning and teaching program of the School. The first step towards commitment to the ongoing curriculum review process was the establishment of an academic position, the Preclinical Curriculum Coordinator (PCC), to lead and coordinate the review process. The PCC works with all the stakeholders to ensure continuity and consistency of the preclinical curriculum review; formulating and articulating a plan for ongoing cyclical preclinical curriculum reviews in the medical program, seeking approval from relevant school committees and updating this plan from time to time as the curriculum evolves, liaising with the evaluation team to ensure the appropriate evaluation of the curriculum has taken place that is necessary to inform curriculum review and development processes, organising all aspects of reviews, including scheduling and chairing meetings and maintaining records of review outcomes, being responsible for referring all outcomes of curriculum review to the Curriculum Committee as necessary, overseeing timely implementation of curriculum developments, working closely with the Medical Education Support Unit (MEDSU), Assessment and Curriculum Committees to ensure the academic integrity of the curriculum, and prepare necessary reports regarding curriculum review and development in the preclinical years as periodically required for accreditation.

The first challenge of the PCC was to devise a strategy to establish cyclical (i.e., ongoing) and coordinated curriculum review in such a way that allowed achievable goals. Reflecting on our own curriculum structure across the first two years (PBLs organised in ‘blocks’ of several weeks duration, with an emphasis on spiral learning), and recognising that the review of the curriculum is not carried out in isolation but along with other learning and teaching activities, reviewing the curriculum ‘blocks’ appeared to provide the most strategic approach i.e., block by block, or grouping blocks related by discipline (e.g., respiratory medicine) from years 1 and 2. This also provided the opportunity to prioritise the areas within the curriculum according to a number of criteria, such as, staff and students’ feedback received in the previous years and performance data. The example we wish to present in this paper for the purposes of curriculum review is the ‘Homeostasis Block’ which is the first block of the preclinical curriculum. The key concepts that are introduced in this three-week block are critical as they form the foundation for learning more advanced concepts in subsequent blocks. This block was given priority in the curriculum review process, based on the information collected from aforementioned sources that emphasized the challenges of learning core concepts in this content heavy and overloaded block. In addition, poor alignment of the resources and assessment items appeared to have a significant effect on learning as well.

The proposed systematic process for the ‘block review’ described in this paper is in consistent with the six key elements identified by Kern (2009) as critical to the process of successful curriculum change: namely, collaboration, communication, implementation, evaluation and feedback and dissemination.

As a principle, for each ‘block review’ these steps were followed with an approximate time frame projected for the full process. As anticipated, the duration taken for reviewing a block varied due to a number of reasons with Homeostasis block taking three months to complete its review cycle. For contrast, we also reviewed the combined respiratory blocks in the preclinical curriculum (a total of 2 PBLs/ teaching weeks from year 1 and a total of 3 PBLs/ teaching weeks from year 2), and this process took 5 months.

## Collaboration and Communication

The first step of reviewing the Homeostasis Block was to identify the team membership to be engaged in the review process. We identified two essential teams that would contribute to the review process: an ‘internal’ team and an ‘external’ (or ‘content expert’) team. The ‘internal team’ (comprised of the domain chairs, representative from the MEDSU, year coordinators, PBL Director, Preclinical Assessment Coordinator), was responsible for mapping the curriculum. This team was expected to remain consistent in its membership throughout the process of curriculum review and development. It was anticipated that by having the same membership, the team would ensure the integrity of the preclinical curriculum (i.e., that change in one block was considered in the context of a two-year preclinical curriculum); it was also anticipated that this team would continue to develop skills in identifying strategies over time to further improve the efficiency of the review process.

The other team identified was the ‘content expert team’, a small number of discipline experts (clinicians, clinical scientists) who were affiliated with the SOMF and committed to improving the preclinical curriculum. We recognised that their input as pivotal in driving the preclinical curriculum towards developing necessary skills, knowledge and attitudes required to master the clinical curriculum in third and fourth year of the medical program. It was anticipated that the membership of the team would change for subsequent block reviews, according to the discipline area being reviewed. The content expert team remains invaluable in ensuring that the curriculum is up-to-date and reflects the current requirements of the field, given that we do not have a standardised curriculum or a Medical Licensing Assessment (MLA) for medical graduates in Australia.

## Implementation

The main focus of this phase was to ensure the content is correct, appropriate to the year level and stage of the curriculum and reflect the current knowledge and practices. This was closely followed by steps to review the constructive alignment between the learning objectives (LOs), resources and assessment items ( Biggs and Tang 2011). One of the challenges to construct and analyse the curriculum map to review the constructive alignment and content was the management of the vast amount of data that needed to be incorporated into the curriculum map ( Lai et al. 2012). In attempting to identify a strategy for this time consuming and complex task, we decided to utilise the web-based curriculum mapping tool Prudentia ^©^ that has been developed by the SOMF ( Steketee 2015). This application not only allowed us to build the curriculum map efficiently and accurately, but also provided the opportunity to explore areas for horizontal and vertical integration across preclinical- and clinical curriculum. The advantage of having a tool that allows tracking the integration was important to overcome the limitation of potential compartmentalisation of content in a block for the review process. The layout of Prudentia ^©^ represent the hierarchical organisation of outcome statement of the SOMF ( Figure 1) which significantly contributed to the accuracy and productivity of manging the metadata. The constructed curriculum map was reviewed by the internal and external teams and after several rounds of meetings a refreshed curriculum map was produced. Broadly the changes were in the form of deleting redundant LOs, adding LOs to cover the areas that were not covered but deemed essential, correcting the wording to reflect the objective of the task, sequencing the order of the LOs appropriately.

After obtaining the endorsement from the Curriculum Committee the curriculum map was directed to the next step of the implementation phase which is a series of parallel tasks that facilitate the constructive alignment and resource delivery. As the key vehicle of curriculum delivery is the PBL tutorials, the tutorials were reviewed with reference to the revised curriculum map. The main objectives of this process were as follows:

• To identify that the PBL scenarios of the block foster identification of LOs (PBL mapping)
• To identify that the clinical and scientific content reflect the current knowledge and practices.
• To identify the areas of better integration of domains
• To identify that the tutors are given adequate information with regard to the PBL scenario to facilitates discussion during the PBL tutorial sessions.

This led to modification of the information in the PBL scenarios and the tutor guides providing better learning opportunities to the students, and better support for our expert small group facilitators. It was crucial to commence the PBL mapping process with the ‘endorsed’ revised curriculum map so that the PBL scenarios remain refreshed as guided by current LOs, however, any ideas and constrains identified while carrying out the PBL review process were fed back to the internal team for further consideration. Maintaining good and efficient communication between key stake holders and clearly identifying roles of key players were essential in successful completion of multiple processes that occurred simultaneously.

The Preclinical Assessment Coordinator also initiated the process of aligning the formative and summative assessment items to the revised curriculum map to foster constructive alignment while the PCC aligned the resources to the map. The use of Prudentia ^®^ significantly improved the efficiency of this task. Resource allocation was further explored by the domain chairs to identify the relevance and adequacy of the resources with reference to the revised curriculum map before disseminating this information for formulating the time tables. Another challenge surfaced while attempting to align the resources was the different Learning Management System (LMS) operated by our partner institution, Murdoch University, to disseminate information pertaining to the course material to the students. Taking the students’ feedback on this issue into account, all the titles of the resources were made consistent in different platforms allowing students to clearly identify the constructive alignment. This would be an area for further exploration with the aim of developing a strategy to either streamline the live systems so that changes in one will automatically modify the other or, to work towards using one system in both academic institutions at least for the medical curriculum.

At the end of this review process internal and external teams were able to produce a revised curriculum map of the block, having defined the content, LOs, timetables, appropriate teaching, learning and assessment methods that utilised relevant and available resources. All the clearly labelled documents produced in the review process were saved in a shared folder of the SOMF data base system that allowed access by key stakeholders of the process. The PCC ensured that the information from teams were collated, updated and stored appropriately. At the end of reviewing each block, a report was prepared outlining the purpose and objectives of reviewing the block, the revised curriculum maps tracking the changes made to the pre-review map, changes to the time tables, changes to the PBL tutorials and further recommendations for consideration in the future. This comprehensive report would be beneficial for the future staff to understand the history behind the curriculum changes at SOMF and for the accreditation purposes.

## Evaluation and Feedback

Evaluation and feedback closes the loop in the curriculum development cycle ( Kern 2009). While data from many potential sources are of importance in the planning phase of the block review, it is equally important to subsequently evaluate the reviewed block with regard to the content, delivery and assessment ( Laskaratos et al. 2014). One limitation of the evaluation is that a comparison of student’s feedback on the block pre- and post-review would not necessarily inform the progress of the content and delivery as it would be different cohorts of students exposed to the pre- and post-reviewed blocks. Nevertheless, using standard instruments, students feedback was evaluated, along with the feedback from the PBL facilitators and the assessment performance data. The necessary evaluation was carried out in accordance with the Evaluation Plan governed by the Evaluation Committee of the SOMF.

## Dissemination

Dissemination of curriculum related work is important for ongoing curriculum development through collaboration and feedback ( Kern 2009). While, the medical curriculum is unique to a particular institution, curriculum review process would share similar objectives. We believe that sharing the information will lead to obtain valuable feedback from curriculum developers to further enhance our review process and will also, assist groups who have similar perspectives.

## Conclusion

In summary, the framework we have discussed in this paper to review the curriculum is based on recommended methods of curriculum review. We intend to use this framework as the basis for our continuous curriculum review. With careful planning and identifying strategies for potential challenges contributed significantly to successfully achieve the intended goals.

## Take Home Messages

• Continuous review of the medical curriculum is critical.
• Developing a framework for continuous curriculum review is challenging.
• Careful planning and identifying strategies are the keys to effectively utilise the limited resources.

## Notes On Contributors

Manori Amarasekera, MBBS, MPhil, PhD, Grad Cert HPE, Preclinical Curriculum Coordinator, School of Medicine Fremantle, The University of Notre Dame Australia, Fremantle, Australia.

Paul S. Noakes, BSc(Hons), PhD, School of Medicine, Year One Coordinator and former Preclinical Curriculum Coordinator, School of Medicine Fremantle, The University of Notre Dame Australia, Fremantle, Australia.

Brian D. Power, BMedSci(Hons) MBBS, PhD, FRANZCP, Cert Psychiatry Old Age, Assistant Dean (Preclinical), School of Medicine Fremantle, The University of Notre Dame Australia, Fremantle, Australia.
