# Supplementary material for: Reviewing the preclinical curriculum in a Problem Based Learning driven medical program: challenges and strategies
Source: MedEdPublish (2016). 2019 Jan 9;8:5. [Version 1] doi: 10.15694/mep.2019.000005.1 (PMC10712617; doi:10.15694/mep.2019.000005.1)
Supplement: Peer review history

# Reviewer report: Reviewer response for version 1

**DOI:** 10.21956/mep.19746.r29611

**Published:** 2019-03-20

**Authors:** Trevor Gibbs

**Affiliations:** 1 AMEE

This review has been migrated. The reviewer awarded 3 stars out of 5 An interesting paper to read which provided some important insights into the construction of an integrated integrated curriculum. I thought that the authors demonstrated some useful aids to curriculum design and used Kern's work to their advantage. I did feel however that the paper tapered of somewhat in the section related to evaluation and feedback. I would have thought that this aspect of any curriculum development was essential- did it / has it worked. I feel that development of this section would have provided more interest to those reading the paper.

Reviewer Expertise:

NA

No decision status is available

# Reviewer report: Reviewer response for version 1

**DOI:** 10.21956/mep.19746.r29612

**Published:** 2019-01-09

**Authors:** Sateesh Babu Arja

**Affiliations:** 1 Avalon University School of Medicine

This review has been migrated. The reviewer awarded 4 stars out of 5 Thank you for asking me to review this paper. I congratulate the university and the authors to take such a meticulous process to review the curriculum and your efforts are appreciated. At our university also, we have similar kind of preclinical curriculum in the first two years. Knowledge, skills, and attitudes are emphasized. Horizontal integration and vertical integration along with PBL sessions are implemented. It is very interesting to see various levels of learning objectives and outcomes. The authors clearly explained the curriculum review process citing six key elements identified by Kern namely, collaboration, communication, implementation, evaluation and feedback, and dissemination. This article is useful to the academic leaders and curriculum reviewers. Thank you.

Reviewer Expertise:

NA

No decision status is available
